# Draft Genome Sequence of *Mycobacterium setense* CSUR47

**DOI:** 10.1128/genomeA.01415-17

**Published:** 2018-01-18

**Authors:** Amar Bouam, Anthony Levasseur, Michel Drancourt

**Affiliations:** a Faculté de Médecine, Aix Marseille Université, URMITE, UMR CNRS 7278, IRD 198, INSERM 1095, Marseille, France

## Abstract

*Mycobacterium setense* CSUR47 is a rapidly growing *Mycobacterium* species strain isolated from pus collected from a left maxillary sinus in Marseille, France. Here, we report the complete 6,278,097-bp genome sequence of *M. setense* CSUR47, which exhibits a 66.40% GC content and encodes 5,863 protein-coding genes, 48 tRNAs, and 9 rRNAs.

## GENOME ANNOUNCEMENT

*Mycobacterium setense* is a nontuberculous, rapidly growing *Mycobacterium* species belonging to the *M. fortuitum* complex of mycobacteria (1). This emerging species was initially isolated from a 52-year-old patient presenting with soft tissue infection and osteitis in France (1). In 2008, a second isolate named *M. setense* CSUR47 was isolated in Marseille from pus collected from the left maxillary sinus of a patient with a history of sinus osseous graft (2). We sequenced and analyzed the whole-genome sequence of strain CSUR47 in order to describe its genomic content and to determine its phylogenetic relationships for facilitating its identification.

*M. setense* CSUR47 was cultured on Middlebrook 7H11 agar supplemented with 10% (vol/vol) oleic acid-albumin-dextrose-catalase (Becton, Dickinson, Sparks, MD, USA). Genomic DNA was then sequenced with MiSeq technology (Illumina, Inc., San Diego, CA, USA) using a mate pair library. The index representation for *M. setense* CSUR47 was determined to be 10.10%. A total of 1,538,022 paired-end reads were filtered per the read qualities and then assembled using the Velvet tool (3). Contigs were combined using SSPACE (4) assisted by manual finishing and GapFiller (5). This yielded a 6,278,097-bp draft genome with a 66.40% GC content and 19 scaffolds composed of 63 contigs. Prodigal (6) was used with default parameters to predict open reading frames (ORFs). Functional annotation was achieved using a BLASTp search against the GenBank (7) and Clusters of Orthologous Groups (COGs) databases (8). When no hit was found, a second search was performed against the NR database using BLASTp. RNAmmer (9), ARAGORN (10), Rfam (11), Pfam (12), and Infernal (13) were used to predict noncoding genes and miscellaneous features. Of the 5,918 predicted genes, 5,863 were protein-coding genes and 55 were RNAs, including 3 5S rRNAs, 3 16S rRNAs, 3 23S rRNAs, and 48 tRNAs. A total of 4,507 genes (76.87% of protein-coding genes) were assigned a putative function, 94 genes were identified as ORFs with no homologs (ORFans) (1.60%), and 982 genes (16.75%) were annotated as hypothetical proteins.

Based on 16S rRNA gene sequence proximity, genomes were selected and incorporated with *in silico* DNA-DNA hybridization (DDH) (14). The DDH values were calculated using the GGDC version 2.0 online tool (15). This analysis yielded sequence similarities of 99.80% with the type strain *M. setense* DSM 45070 (GenBank accession number JTJW00000000), 89.80% with the nonpathogenic strain Manresensis (JTLZ00000000), 30.50% with *M. farcinogenes* DSM 43637 (CCAY000000000), 30.40% with *M. senegalense* CK2 (LDPU00000000), 29.70% with *M. houstonense* ATCC 49403 (NZ_FJVO00000000), 29.40 with *M. fortuitum* DSM 46621 (ALQB00000000), and 20.60 with *Mycobacterium gilvum* PYR-GCK (NC_009338). These results confirmed that strain CSUR47, also known as isolate 74023791 (2), belongs to the *M. setense* species within the *M. fortuitum* complex. Furthermore, *M. setense* CSUR47 was more closely related to the clinical *M. setense* DSM 45070 type strain than to the environmental Manresensis strain isolated from Cardener River in Manresa, Spain, and was shown to be less virulent than *M. bovis* BCG in immunocompromised mice (16).

### Accession number(s).

The *M. setense* CSUR47 genome sequence has been deposited at EMBL under the accession number OEJY01000000.
